# Pregnancy planning and acceptance and maternal psychological distress during pregnancy: results from the National Perinatal Survey, France, 2016

**DOI:** 10.1186/s12884-022-04496-3

**Published:** 2022-02-28

**Authors:** Caroline Moreau, Camille Bonnet, Maxime Beuzelin, Béatrice Blondel

**Affiliations:** 1 Department of Population, Family and Reproductive Health, Johns Hopkins Bloomberg School of Public Health, 615 N Wolfe Street, Baltimore, MD 21203 USA; 2 Soins et Santé Primaire, CESP Centre for Research in Epidemiology and Population Health, U1018, INSERM, F-94805 Villejuif, France; 3 Obstetrical, Perinatal and Pediatric Research Team, INSERM, Université de Paris, F-75014 Paris, France

**Keywords:** Pregnancy intentions, Pregnancy acceptability, Pregnancy planning, Maternal psychological health, Maternal depressive symptoms

## Abstract

**Background:**

Studies report heightened risks of mental health problems among women who experience an unintended pregnancy, but few consider the complexity of pregnancy intentions. In this study, we evaluate how different dimensions of pregnancy intentions (pregnancy planning and pregnancy acceptance) relate to two maternal depressive symptoms and perceived psychological distress.

**Methods:**

This study draws from a cross-sectional national survey conducted in all maternities in France over a one-week period in 2016. All mothers 18 years and older who had a live birth during the study period were invited to participate. After excluding women who underwent infertility treatment, our analytical sample included 10,339 women. We first described levels and correlates of pregnancy planning and acceptance, defined in four categories; planned/welcomed, unplanned/welcomed, planned/unwelcomed, unplanned/unwelcomed. We then assessed the bivariate and multivariate associations between pregnancy planning and acceptance and two outcomes: women’s self-perceived psychological health and the presence of two depressive symptoms during pregnancy. We used multivariate logistic regressions to evaluate these associations, after adjusting for socio-demographic and medical factors.

**Results:**

Altogether 7.5 to 24.1% of mothers perceived their psychological health during pregnancy was poor, according to pregnancy planning and acceptance categories and 10.3 to 22.4% indicated feelings of sadness and loss of interest during pregnancy, according to pregnancy planning and acceptance categories. As compared to women with planned/welcomed pregnancies, the odds of perceived poor psychological health and depressive symptoms were 2.55 times (CI 2.20–2.95) and 1.75 times higher (CI 1.51–2.02), respectively, among unplanned/unwelcomed pregnancies and 2.02 (CI 1.61–2.53) and 2.07 (CI 1.7–2.5) higher, among planned/unwelcomed pregnancies. Among women with unplanned pregnancies, we also found higher odds of perceived poor psychological health among women whose pregnancy was unwelcomed while the odds of depressive symptoms were not different by pregnancy planning status among women with unwelcomed pregnancies.

**Conclusions:**

These findings consolidate previous reports of the association between pregnancy intentions and maternal psychological distress, while further specifying the relationship, which mostly depends on the acceptance of pregnancy timing rather than on pregnancy planning. Identifying women with low pregnancy acceptance can potentially enhance current medical practice by improving early detection of maternal depression.

## Background

Despite widespread contraceptive coverage in high-income countries, the proportion of pregnancies that are unintended ranges from 34% in the Unite Kingdom [1], to 36% in France [2] and 46% in the United States [3]. Unintended pregnancies represent a significant public health concern due to associated increases in maternal and perinatal morbidity [4].

Recent attention has concentrated on the association between pregnancy intentions and mental health, as poor mental health is suggested to be a cause and consequence of unintended pregnancy. Some studies indicate that depressive symptoms affect contraceptive choices and practices [5], which determine unintended pregnancy risk [6, 7]. Other studies suggest experiencing an unintended pregnancy is likely to cause stress, which may contribute to heightened risk of poor mental health [8]. Two meta-analyses reported an increase in the prevalence of antepartum depression among women who experienced an unintended birth [9, 10]. While the causal pathways linking pregnancy intentions to poor psychological health are complex, the implications are profound for mothers and infants given the associations between maternal depression and other behaviors, such as substance use [11], and the consequences on perinatal health and child development [12, 13].

A growing body of work has questioned the relevance of the dichotomous indicator of unintended pregnancy, defined as the combination of unwanted and mistimed pregnancies [14–16]. A systematic review supports the use of more nuanced indicators, showing the risk of maternal depressive symptoms is greater among women who have an unwanted birth compared to a mistimed birth [10]. Likewise, Gariepy et al.’s prospective study in the US suggests that the acceptance of the timing of pregnancy is a better predictor of mental health than initial pregnancy plans [17]. Similar results were reported in a Swedish study [18], while a network analysis exploring the co-occurrence of maternal psychosocial risks among human immunodeficiency virus (HIV) positive women indicates that distress about pregnancy rather than intention was the most central feature in a network of psychosocial risks and was strongly related to antenatal depression [19].

These findings support Aiken et al.’ framework focusing on pregnancy acceptability as a salient measure to inform maternal health [16]. The ways in which women’s perspectives on pregnancy relate to their psychological health during pregnancy deserves further consideration, as the acceptability of a pregnancy is tied to economical or relational circumstances that also affect health processes [4]. Building on this work, we investigate the intersecting contribution of pregnancy planning and acceptance on antenatal psychological distress, in the form of perceived psychological health and two depressive symptoms, among women giving birth in France in 2016.

## Material and methods

This study draws from the 2016 French National Perinatal Survey [20]. The survey included all mothers who gave birth in France over a one-week period. Women were invited to participate in the study after delivery, during their maternity admission and were interviewed by trained midwives who collected information about their sociodemographic characteristics, pregnancy intentions and contraceptive behaviors at the time of conception, and their health and behaviors during pregnancy, including care-seeking behaviors. The survey included additional self-administered questions at the end of the interview. Medical information related to maternal health was extracted from medical health records.

### Study population

A total of 13,147 women delivered during the survey implementation and 12,964 were eligible (women younger than age 18 (*n* = 56) and women who delivered a stillbirth were not eligible). A total of 1202 eligible women were not interviewed: 543 refused participation, 245 did not speak French, 144 suffered serious medical complications for themselves or their child, and 79 left the facility before the scheduled interview. For this analysis, we further excluded 806 women who underwent infertility treatment. Finally, we excluded 394 women with missing information about pregnancy planning or acceptance and 23 to 223 women with missing information on one of the psychological health outcomes. Our final analytical sample comprised between 10,339 and 10,539 women.

### Measures

**Fig. 1:**
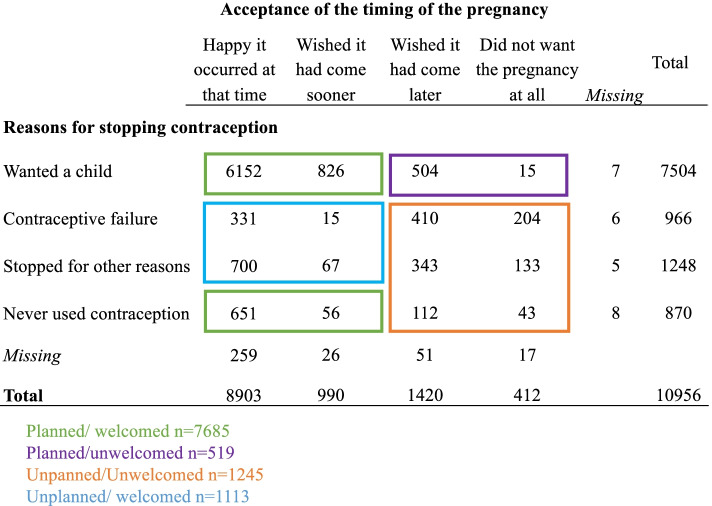
Construction of the combined measure of pregnancy acceptance and planning

We used a proxy measure of women’s planning and acceptance of pregnancy as a combination of pre-conception contraceptive behaviors and women’s reaction to the timing of the pregnancy. Specifically, we assessed pregnancy planning as a function of women’s contraceptive behavior prior to pregnancy with response options distinguishing between women who ceased contraception to become pregnant (planned pregnancy) from those who stopped following a contraceptive failure or for another reason (unplanned pregnancy). Women were also asked about their reaction to the timing of the pregnancy with the following response options: “happy to be pregnant now”; “would have liked to be pregnant sooner”; “would have liked to be pregnant later”; and “did not want to be pregnant at all”. Combining the reported contraceptive behaviors and women’s acceptance of the timing of the pregnancy, we categorized women into four categories of pregnancy planning and acceptance: planned/welcomed, planned/unwelcomed unplanned/welcomed, and unplanned/unwelcomed (Fig. 1).

We considered the following sociodemographic factors: age, parity, cohabitation status, education, household income, type of health insurance, country of birth and history of abortion. Women’s chronic medical conditions prior to pregnancy (ie., diabetes, hypertension, HIV, or any other chronic diseases requiring preconception care) were extracted from the medical records and combined in a single measure. These conditions, however, did not include antecedents of mental health illness.

The main mental health distress outcomes were assessed using two indicators: women’s self-assessment of their psychological health during the pregnancy, and two depressive symptoms during two consecutive weeks in pregnancy. Specifically, women were asked to assess their psychological health during the pregnancy (response options included good, fairly good, not very good, and bad). They subsequently self-completed two questions corresponding to a modified version of the Patient health Questionnaire 2 (PHQ2) indicator [21] about feelings of sadness, depression or hopelessness, and about loss of interest in most things, such as leisure activities. Unlike the PHQ2 questions assessing the frequency of symptoms over the last 2 weeks, our questions examined the presence or absence of these symptoms over two consecutive weeks during pregnancy. Thus, we were unable to calculate the PHQ2 score ranging from 0 to 6 based on frequency of each symptom [21] but instead, constructed a binary measure of depressive symptoms based on the presence of both symptoms for two consecutive weeks during the course of the pregnancy. We also defined an indicator assessing the presence of both depressive symptoms and perceived poor psychological health. Finally, to examine care-seeking behaviors, women were asked if they had consulted a health professional (medical doctor, psychologist or psychotherapist) for psychological reasons during the pregnancy.

### Statistical analyses

We first described pregnancy planning and acceptance according to women’s sociodemographic characteristics and conducted a multinomial logistic regression model. We then estimated the prevalence of women’s perceived psychological health and the prevalence of experiencing two depressive symptoms during two consecutive weeks in the course of the pregnancy. We examined the sociodemographic and reproductive health factors related to these psychological distress measures.

Next, we conducted a series of multivariate logistic regressions to evaluate the associations between our four-category measure of pregnancy planning/acceptance and each psychological distress outcome (perceived psychological health, two depressive symptoms and care seeking behaviors), after adjusting for sociodemographic and medical factors. We also conducted two sub-analyses, first comparing psychological distress outcomes according to pregnancy acceptance among women who reported their pregnancy was unplanned and then comparing psychological distress outcomes according to pregnancy planning among women who reported their pregnancy was unwelcomed.

## Results

Altogether 72.8% of births were planned and welcomed, 4.9% were planned but unwelcomed, 10.5% were unplanned but welcomed and 11.8% were unplanned and unwelcomed (Table 1).

**Table 1:** Socioeconomic and medical factors according to pregnancy planning and acceptance among women giving birth in France in 2016

	Pregnancy planning/acceptance					
	*Total (%)*	Planned/ welcomed	Planned / unwelcomed	Unplanned/ welcomed	Unplanned/unwelcomed	*p-value*
		**7685 (72.8)**	**519 (4.9)**	**1113 (10.5)**	**1245 (11.8)**	
**Mother age (in years)**						< 0.001
18–19	154 (1.5)	0.9	1.4	2.1	4.4	
20–24	1260 (11.9)	10.2	8.8	15.5	20.6	
25–29	3435 (32.5)	33.0	36.4	29.2	30.9	
30–34	3604 (34.1)	35.9	35.5	29.1	27.2	
35–39	1741 (16.5)	16.7	16.2	18.2	13.6	
≥ 40	368 (3.5)	3.3	1.7	5.9	3.3	
**Parity**						< 0.001
0	4297 (40.7)	42.0	38.5	42.4	32.0	
1	3881 (36.8)	39.4	39.1	29.5	25.9	
2	1576 (14.9)	13.3	16.8	15.6	23.5	
≥ 3	804 (7.6)	5.3	5.6	12.5	18.6	
**Living with a partner**						< 0.001
Yes	9636 (91.4)	94.8	93.6	83.8	76.0	
No	907 (8.6)	5.2	6.4	16.2	24.0	
**Country of birth**						< 0.001
France	8560 (81.1)	82.2	83.1	79.8	74.2	
Other European country	413 (3.9)	3.9	4.2	5.3	2.9	
North African	764 (7.2)	7.1	4.2	6.7	9.5	
Other African country	493 (4.7)	3.7	4.6	5.2	10.0	
Other country	331 (3.1)	3.1	3.9	3.0	3.4	
**Level of education**						< 0.001
< high school	2456 (23.4)	20.2	21.4	31.0	37.5	
High school or technical degree	2293 (21.9)	20.6	22.8	23.5	28.1	
1–2 years > high school	2003 (19.1)	19.8	20.9	17.6	15.8	
3 years > high school	1893 (18.1)	19.8	17.2	14.6	10.9	
5 years + > high school	1831 (17.5)	19.7	17.7	13.3	7.7	
**Health coverage at beginning of pregnancy**						< 0.001
National health insurance	8983 (85.2)	88.6	85.7	80.9	67.5	
Insurance for low income/ undocumented or none	1565 (14.8)	11.4	14.3	19.1	32.5	
**Monthly household resources**						< 0.001
< 1000 euros	1016 (9.8)	7.3	7.1	13.9	22.4	
1000–1499 euros	913 (8.8)	7.2	6.9	12.7	15.9	
1500–2000 euros	1332 (12.8)	11.9	12.9	15.9	16.0	
1999–3000 euros	2917 (28.1)	29.4	32.0	26.8	25.8	
2999–4000 euros	2394 (23.1)	25.4	24.1	17.3	13.4	
4000 euros or more	1808 (17.4)	19.8	17.0	13.4	6.5	
**History of abortion**						< 0.001
0	8638 (83.3)	86.0	80.8	75.0	74.8	
1	1334 (12.9)	11.2	15.1	18.2	17.7	
2 or more	400 (3.8)	2.8	4.1	6.8	7.5	
**Pre-pregnancy chronic conditions**^**a**^						< 0.001
yes	260 (2.5)	2.1	1.7	4.4	3.5	
no	10,260 (97.5)	97.9	98.3	95.6	96.5	

^a^Chronic conditions prior to pregnancy include diabetes, chronic hypertension, HIV, or any other chronic pathologies excluding mental health illness

### Sociodemographic factors related to pregnancy planning and acceptance

Women with unplanned pregnancies were younger, less likely to be cohabiting, less educated, and more likely to be low income and receive government health insurance for low-income individuals than women with planned pregnancies. These sociodemographic differences were particularly evident for women who had unplanned and unwelcomed pregnancies, with women in this category more likely to have migrated from Sub-Saharan Africa (Table 1). Women with unplanned pregnancies were also more likely to include the following factors: higher parity, a history of abortion and a pre-pregnancy condition that heightened their risk of maternal morbidity. Multivariate analysis supported most of the previous associations indicating fewer differences between planned/welcomed and planned/unwelcomed pregnancies than between planned/welcomed and unplanned/welcomed pregnancies (Table 2).

**Table 2:** Maternal characteristics associated with pregnancy planning and acceptance among women giving birth in France in 2016: results from multivariate analysis

	Planned/unwelcomed vs Planned/welcomed		Unplanned/welcomed vs Planned/welcomed		Unplanned/unwelcomed vs Planned/welcomed	
	aOR	95% CI	aOR	95% CI	aOR	95% CI
**Age**						
18–24	0.93	0.65–1.34	1.48	1.17–1.87	3.05	2.44–3.81
25–29	1.12	0.89–1.40	1.08	0.90–1.28	1.50	1.25–1.79
30–34	1		1		1	
35–39	0.92	0.69–1.21	1.17	0.96–1.43	0.75	0.60–0.93
≥ 40	0.55	0.28–1.10	1.61	1.17–2.22	0.69	0.47–1.02
**Parity**						
0	1		1		1	
1	1.08	0.87–1.34	0.80	0.68–0.94	1.16	0.97–1.38
2	1.32	0.98–1.77	1.16	0.94–1.44	3.51	2.86–4.29
≥ 3	1.09	0.69–1.74	1.91	1.46–2.49	6.16	4.78–7.94
**Live with a partner**						
Yes	1		1		1	
No	1.22	0.79–1.88	2.51	2.00–3.16	3.25	2.64–4.00
**Country of birth**						
France	1		1		1	
Other	0.90	0.69–1.18	0.94	0.78–1.12	1.11	0.94–1.32
**Level of education**						
< high school	0.93	0.65–1.34	1.20	0.93–1.57	1.09	0.81–1.45
High school or technical degree	1.07	0.77–1.49	1.14	0.88–1.47	1.25	0.95–1.66
1–2 years > high school	1.04	0.76–1.43	1.08	0.84–1.39	1.16	0.88–1.55
3 years > high school	0.90	0.65–1.23	0.98	0.76–1.25	1.02	0.76–1.37
5 years or more > high school	1		1		1	
**Health coverage at beginning of pregnancy**						
National health insurance	1		1		1	
Insurance for low income/ undocumented or none	1.56	1.09–2.23	0.92	0.73–1.15	1.18	0.97–1.44
**Monthly household resources**						
< 1000 euros	0.77	0.44–1.33	1.40	0.99–2.00	2.31	1.60–3.34
1000–1499 euros	0.81	0.50–1.33	1.49	1.08–2.04	2.07	1.46–2.92
1500–2000 euros	1.09	0.74–1.61	1.37	1.03–1.81	1.90	1.38–2.61
1999–3000 euros	1.19	0.87–1.63	1.15	0.90–1.46	1.69	1.26–2.25
2999–4000 euros	1.05	0.78–1.43	0.94	0.74–1.20	1.33	0.99–1.78
4000 euros or more	1		1		1	
**History of abortion**						
0	1		1		1	
1	1.45	1.12–1.88	1.74	1.46–2.08	1.54	1.29–1.85
2 or more	1.57	0.98–2.50	2.15	1.61–2.88	2.11	1.59–2.80
**Chronic pre-pregnancy medical conditions**^**a**^						
Yes	0.78	0.38–1.60	2.05	1.45–2.89	1.66	1.14–2.41
No	1		1		1	

^a^Chronic conditions prior to pregnancy include diabetes, chronic hypertension, HIV, or any other chronic pathologies excluding mental health illness

### Maternal psychological distress and health care seeking

Altogether, 24.0% of women reported feeling sad, depressed or hopeless, and 15.5% indicated a loss of interest in most things, for at least two consecutive weeks of pregnancy, while 12.5% presented both depressive symptoms (Table 3). They are described in the remainder of the article as presenting with depressive symptoms during pregnancy. In addition, 10.1% of mothers perceived they had poor psychological health during pregnancy. Altogether, 4.5% reported depressive symptoms and perceived poor psychological health.

**Table 3:** Women’s psychological health during pregnancy according to their socioeconomic characteristics among women giving birth in France in 2016

	Poor psychological health (self-assessed)	Sadness for 2 consecutive weeks	Loss of interest for 2 consecutive weeks	Depressive symptoms (sadness & loss of interest)	Poor psychological health & depressive symptoms
**Total**	**10.1**	**24.0**	**15.5**	**12.5**	**4.5**
**Mother age (in years)**	*0.0033*	*< 0.0001*	*< 0.0001*	*< 0.0001*	*0.0004*
18–19	11.1	28.5	24.5	15.9	7.3
20–24	10.8	30.6	24.8	16.6	5.7
25–29	9.3	23.1	18.7	11.9	4.0
30–34	9.7	21.9	16.1	10.9	3.8
35–39	10.7	24.4	17.6	13.2	5.3
≥ 40	15.9	27.9	19.4	14.8	7.6
**Parity**	*< 0.0001*	*0.0235*	*0.0002*	*0.0063*	*0.0004*
0	8.2	24.1	19.3	12.7	4.1
1	10.0	23.0	16.5	11.2	4.1
2	12.2	24.3	19.3	13.5	5.5
≥ 3	18	28.1	22.1	15.1	7.0
**Live with a partner**	*< 0.0001*	*< 0.0001*	*< 0.0001*	*< 0.0001*	*< 0.0001*
Yes	9.1	22.3	16.9	11.3	3.9
No	21.7	42.2	27.6	25.1	11.4
**Country of birth**	*< 0.0001*	*< 0.0001*	*< 0.0001*	*< 0.0001*	*< 0.0001*
France	9.1	22.4	16.7	11.0	4.0
Other European country	9.2	22.1	16.7	12.0	4.2
North African	14.6	30.2	26.9	18.4	7.1
Other African country	17.7	41.4	35.7	26.7	9.6
Other country	13.7	28.4	23.1	16.1	4.5
**Level of education**	*< 0.0001*	*< 0.0001*	*< 0.0001*	*< 0.0001*	*< 0.0001*
< high school	13.6	29.4	23.1	15.5	6.1
High school or technical degree	11.7	26.6	20.9	14.5	5.6
1–2 years > high school	10.0	23.2	19.3	11.8	3.8
3 years > high school	8.2	19.5	14.8	9.9	3.7
5 years + > high school	5.9	18.9	12.2	8.8	2.8
**Health coverage at beginning of pregnancy**	*< 0.0001*	*< 0.0001*	*< 0.0001*	*< 0.0001*	*< 0.0001*
National health insurance	8.9	22.4	16.9	11.2	3.9
Insurance for low income/ undocumented or none	16.8	33.4	27.6	19.7	8.1
**Monthly household resources**	< 0.0001	< 0.0001	< 0.0001	< 0.0001	< 0.0001
< 1000 euros	18.8	37.0	29.5	21.6	9.6
1000–1499 euros	13.2	29.8	24.0	16.8	5.5
1500–2000 euros	11.1	25.5	22.3	13.6	5.2
1999–3000 euros	9.5	24.8	18.5	12.9	4.6
2999–4000 euros	8.3	19.4	14.2	9.2	3.1
4000 euros or more	6.3	18.2	12.7	8.3	2.6
**History of abortion**	< 0.0001	< 0.0001	< 0.0001	0.0003	< 0.0001
0	9.3	22.6	17.4	11.7	4.1
1	12.1	28.7	23.2	15.0	5.1
2	18.8	35.8	22.0	16.3	8.6
3 or more	22.2	32.9	27.5	19.0	10.1
**Chronic pre-pregnancy medical conditions**	*0.0692*	*0.07*	*0.0018*	*0.0579*	*0.31*
Yes	13.5	28.7	25.9	16.3	5.8
No	10.0	23.9	18.2	12.3	4.5

^a^Chronic conditions prior to pregnancy include diabetes, chronic hypertension, HIV, or any other chronic pathologies excluding mental health illness

Few women (6.4%) consulted a health professional for psychological problems (Table 4). Women with depressive symptoms were more likely to have consulted (18.6% versus 7.5% for women who did not present both depressive symptoms, *P* < 0.001), although most did not (results not shown). The same was true for women who perceived poor psychological health during pregnancy (19.2% versus 7.9%, *P* < 0.001). Altogether, 28.4% of women who perceived poor psychological health and reported two depressive symptoms had consulted a health professional for psychological problems during the pregnancy.

**Table 4:** Pregnancy planning and acceptance, psychological health and care during pregnancy among women giving birth in France in 2016: results from multivariate analysis

	Poor Psychological health (self-assessed)			Sadness for 2 consecutive weeks			Loss of interest for 2 consecutive weeks			Both depressive symptoms			Depressive symptoms & poor psychological health			Consulted a professional for psychological difficulties		
	%	aOR^a^	95% CI	%	aOR^a^	95% CI	%	aOR^a^	95% CI	%	aOR^a^	95% CI	%	aOR^a^	95% CI	%	aOR^a^	95% CI
All	10.1			24.0			18.5			12.5			4.5			6.4		
Planned/ welcomed	7.5	1		20.6	1		15.7	1	.	10.3	1		3.2	1		5.7	1	
Planned/ unwelcomed	14.9	2.02	1.61–2.53	36.9	1.81	1.59–2.05	27.6	1.73	1.48–2.02	21.6	2.07	1.7–2.5	6.6	2.11	1.47–3.03	10.8	1.95	1.49–2.55
Unplanned / welcomed	10.4	1.25	1.03–1.53	24.5	1.09	0.97–1.23	20.0	1.13	0.99–1.30	12.2	1.03	0.9–1.2	4.3	1.13	0.81–1.57	7.1	1.19	0.94–1.51
Unplanned/ unwelcomed	24.1	2.55	2.20–2.95	39.2	1.63	1.48–1.80	30.7	1.58	1.41–1.77	22.4	1.75	1.51–2.02	11.9	2.97	2.36–3.73	8.3	1.47	1.16–1.85
*Sub-analysis among unplanned pregnancies*																		
Unplanned/welcomed	10.4	1		24.5	1		20.0	1		12.2	1		4.3	1		7.1	1.00	
Unplanned/unwelcomed	24.1	2.00	1.62–2.48	39.2	1.56	1.36–1.78	30.7	1.49	1.27–1.74	22.4	1.79	1.45–2.20	11.9	2.63	1.85–3.72	8.3	1.25	0.92–1.69
*Sub-analysis among unwelcomed pregnancies*																		
Planned/unwelcomed	14.9	1		36.9	1		27.6	1		21.6	1		6.6	1	.	10.8	1	
Unplanned/unwelcomed	24.1	1.22	0.95–1.56	39.2	0.98	0.80–1.14	30.7	1.03	0.86–1.24	22.4	0.97	0.78–1.21	11.9	1.48	1.01–2.16	8.3	0.74	0.52–1.05

^a^Adjusted for maternal age, country of birth, parity, level of education, partner cohabitation, monthly household income, health insurance coverage at the beginning of pregnancy and high risk maternal conditions

Poor perceived psychological health and depressive symptoms were more common among the youngest and oldest mothers, among higher parity women and among women who had a history of induced abortion (Table 3). Women from socially disadvantaged backgrounds, including women who were foreign-born, less educated and lower income, were also more likely to perceive poor psychological health and report depressive symptoms during pregnancy.

### Psychological distress according to pregnancy planning and acceptance

Perceived poor psychological health varied according to pregnancy planning and acceptance, ranging from 7.5% among those who had planned/welcomed pregnancies to 24.1% among those who had unplanned/unwelcomed pregnancies (Table 4). Likewise, feelings of sadness for two consecutive weeks during the course of the pregnancy ranged from 20.6 to 39.2% while loss of interest doubled from 15.7 to 30.7%, according to pregnancy planning and acceptance. Ultimately, having both depressive symptoms ranged from 10.3 to 22.4%. The same increase was noted among women who both perceived poor psychological health and depressive symptoms, rising from 3.2 to 11.9% according to pregnancy planning and acceptance.

### Maternal psychological health according to acceptance of timing of pregnancy

Bivariate results were confirmed in multivariate analysis showing greater odds of perceived poor psychological health, depressive symptoms or both among women with unplanned/unwelcomed pregnancies relative to women with planned/welcomed pregnancies, adjusted odds ratios ranging from 1.58 (CI 1.41–1.77) to 2.97 (CI 2.36–3.73) depending on the outcome (Table 4). Women with planned/unwelcomed pregnancies had elevated odds of perceived poor psychological health compared to women with planned/welcomed pregnancies with adjusted odds ratios ranging from 1.73 (CI 1.48–2.02) to 2.11 (CI 1.47–3.03).

When selecting women who reported unplanned pregnancies, we also found greater odds of perceived poor psychological health across all indicators among women with unwelcomed pregnancies relative to those who had welcomed pregnancies, with adjusted odds ratios ranging from 1.49 (CI 1.27–1.74) to 2.63 (CI 1.85–3.72).

### Maternal psychological distress according to pregnancy planning

In contrast to previous findings, we found no differences in depressive symptoms (aOR = 0.99 (CI 0.82–1.19)) and only slightly elevated odds of perceived poor psychological health (aOR = 1.25 (CI 1.03–1.53)) according to pregnancy planning status among women who had welcomed pregnancies (result not shown). There were no differences in perceived poor psychological health or depressive symptoms reported alone, according to pregnancy planning among women who had unwelcomed pregnancies; although the odds of reporting both were higher when women had an unplanned pregnancy (aOR = 1.48 (CI 1.01–2.16)) (Table 4).

### Care seeking according to pregnancy planning and acceptance

Finally, women with unwelcomed pregnancies were more likely to have consulted a health professional for psychological problems compared to women with welcomed pregnancies, regardless of pregnancy planning status, while women with unplanned/welcomed pregnancy were as likely to consult than women with planned/welcomed pregnancies (Table 4).

## Discussion

Our results show that unplanned births account for almost one in four live births in France, but half are well accepted based on women’s reaction to the timing of the pregnancy. Pregnancy acceptance was strongly related to antenatal psychological symptoms, as women who experienced unwelcomed pregnancies had about twice the odds of reporting perceived poor psychological health and presenting with two depressive symptoms for at least two consecutive weeks during pregnancy, compared to women with planned/welcomed pregnancies, with little effect of planning status, even after adjusting for socio-demographic and medical factors. This increased risk was not observed among women whose unplanned pregnancies were well accepted.

Our findings are consistent with previous reports of the association between pregnancy intentions and maternal mental health distress, while further specifying the relationship which mostly depends on acceptability of pregnancy timing rather than on pregnancy planning. In support of Aiken and al.’s framework [16], we found that women with low pregnancy acceptance were more likely to experience psychological distress compared to women who responded positively to the timing of pregnancy whether planned or not. These results are in line with Lancaster et al. and Abajobir et al.’s systematic reviews, albeit using different pregnancy intention measures [9, 10]. Specifically, our measure of unplanned and unwelcomed pregnancy most closely mimics the association reported between unwanted pregnancies and maternal depression in Abajobir et al.’s meta-analysis [10]. However, our distinction between unplanned/welcomed and unplanned/unwelcomed pregnancy provides additional insights on the importance of women’s reaction to the pregnancy, confirming prior work among smaller convenience samples, suggesting that pregnancy acceptability matters more than planning [17, 18].

While the directionality of the association cannot be established in our study, especially as we have no information about pre-pregnancy mental health history, the relevance of pregnancy acceptability as a marker of risk for maternal mental distress remains critical both from a public health and clinical perspective. This is especially pertinent as depression during pregnancy is a common source of maternal morbidity and is associated with increased risk of postpartum depression [22], and adverse perinatal and child health [12, 13]. A pooled estimated prevalence based on meta-regression data from 101 studies evaluates the prevalence of perinatal depression at 11.4% in high-income countries [23]. This estimate rises to 21% among women with an unintended pregnancy according to Abajobir et al.’s meta-analysis [10]. Thus, monitoring and supporting the health and social needs of women with low pregnancy acceptability who tend to present with depressive symptoms early in pregnancy [24], can enhance medical practice by improving early detection and care of maternal depression. Such interventions are needed to reduce unmet need for maternal psychological support, which could be substantial, as suggested by our study showing that only 28% of women who perceived poor psychological health and reported two depressive symptoms during pregnancy had consulted a healthcare provider for their psychological concerns.

The present study has a number of limitations that should be considered when interpreting the results. The multi-thematic nature of the National Perinatal Survey, destined to monitor key national perinatal indicators and compliance with clinical guidelines, prevented the inclusion of a number of validated multi-item measures to reduce interview time. As an alternative, we assessed pregnancy planning and acceptance using two questions that are routinely discussed during clinical encounters rather than the more comprehensive London Measure of Unintended Pregnancy, which includes six questions [25] . While our indicator of pregnancy acceptance does not capture the complexities of women’s emotional reactions to a pregnancy [16], we nonetheless were able to discern different perspectives on pregnancy that are salient to inform a women-centered approach to pregnancy care. Measurement concerns extend to maternal psychological health, as this secondary analysis did not use diagnostic measures of mental health such as the World Mental Health Composite International Diagnostic Interview (CIDI) v2.1 [26], nor did it use validated measures of depressive symptoms, mostly evaluated using questionnaire scales [27], such as the widely used Edinburgh Postnatal Depression Scale [28] or the Patient health Questionnaire (PHQ9) scale [29] (from which our 2 items of depression were extracted). These scales comprise more items than could be integrated in the Perinatal survey for the same reasons specified for pregnancy intentions measures. In addition, our retrospective measures do not relate to a specific timing during pregnancy and are therefore unable to capture the changing prevalence of antepartum depression [27]. While we acknowledge that these limitations are potential threats to the internal validity of our findings, our assessment of depressive symptoms (combining two modified PHQ2 items) during pregnancy are in the range of maternal depressing symptoms estimates using the PHQ9 measure [29] or mood disorders evaluated using CIDI interviews among postpartum women enrolled in the National Comorbidity Survey [30]. In addition, our reported associations with pregnancy intentions are consistent with previous studies, showing a doubling of the odds of poor psychological health among women with unwanted pregnancy [10]. Our results are also consistent across our indicators, whether we consider the presence of two depressive symptoms, perceived poor psychological health alone, or both. The cross-sectional design and timing of the survey may also introduce recall bias and post-rationalization of pregnancy intentions. Studies have shown that pregnancy acceptability can change, with unintended pregnancies reclassified as intended after the birth of a child [31]. Such reclassification is likely to impact the association between pregnancy acceptability and mental distress if reclassification differs by mental distress status. Temporality of exposure and outcome is also a concern as we were not able to adjust for women’s pre-pregnancy mental health history, which is an important predictor of maternal mental health [32]. These limitations prevent us from making any claims about causality based on our findings.

Despite these limitations, this study adds to the literature, highlighting the importance of women’s acceptance of pregnancy timing as a correlate of maternal mental distress. The study has a number of strengths, including the use of a large representative sample of women delivering a live birth in France and the detailed information about women’s social and demographic background. This sociodemographic information addresses some of the limitations of previous research, allowing adjustments for multiple social confounders of the relation between pregnancy intentions and maternal mental distress. However, confounding may still exist in this observational study, including for example life events happening after the planning of the pregnancy that may affect pregnancy acceptance and women’s mental distress. We believe our results provide empirical evidence of the heterogeneous linkages between pregnancy intentions and maternal health distress, drawing attention to the concept of pregnancy acceptability rather than planning as a critical marker of maternal risk that can be used in clinical practice to tailor healthcare and social services for women at higher risk of experiencing psychological distress during pregnancy. We suggest future research should revisit the association between pregnancy acceptability and antenatal/postpartum depression using a longitudinal design and diagnostic or validated psychometric measures of depressive symptoms to evaluate how pregnancy acceptability at the beginning of the pregnancy, or in the course of pregnancy, relates to and predicts maternal mental health, while also identifying barriers to mental health care, which may be prevalent based on our preliminary results.

## Conclusion

Our findings specify the relationship between pregnancy intentions and maternal psychological health, which mostly depends on the acceptance of pregnancy timing rather than on pregnancy planning. A holistic assessment and response to women’s health and social needs during pregnancy are critical in reducing unmet need for psychological care, which we found to be high in our study and to address social inequalities in women’s pregnancy experiences and outcomes.

## Data Availability

The datasets generated and analyzed for the current study could not be publicly available due to individual privacy of the study participants but are available from the principal investigator Béatrice Blondel upon reasonable request.

## Abbreviations

HIV: Human immunodeficiency virus
PHQ9: Patient health Questionaire-9
PHQ2: Patient health Questionaire-2
CES-D: Center for Epidemiologic Studies- Depression
